# 5-HT_3_ Receptor MX Helix Contributes to Receptor Function

**DOI:** 10.1021/acschemneuro.2c00339

**Published:** 2022-07-22

**Authors:** James Mocatta, Susanne M. Mesoy, Dennis A. Dougherty, Sarah C. R. Lummis

**Affiliations:** † Department of Biochemistry, University of Cambridge, Tennis Court Road, Cambridge CB2 1GA, United Kingdom; ‡ Division of Chemistry and Chemical Engineering, California Institute of Technology, Pasadena, California 91125, United States

**Keywords:** Cys-loop receptor, binding site, mutagenesis, noncanonical amino acid, nonsense suppression

## Abstract

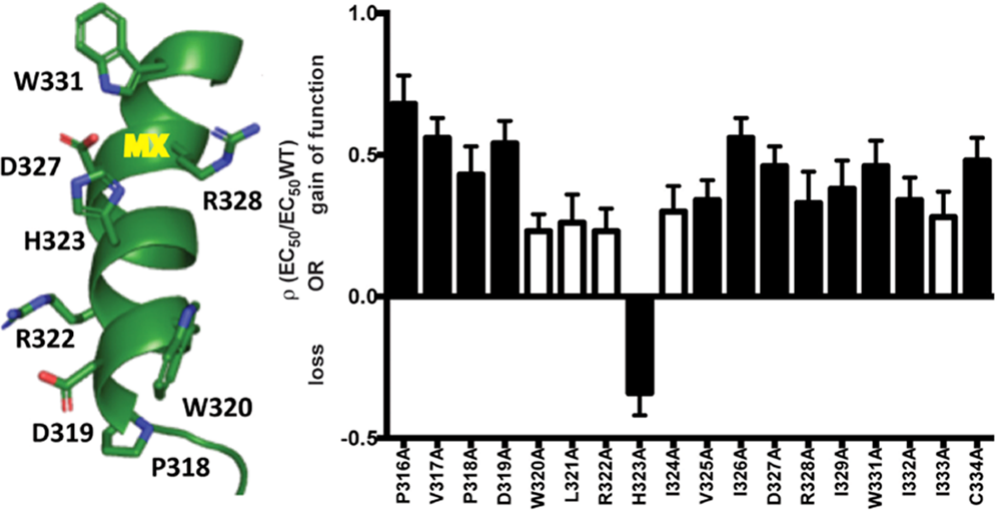

5-HT_3_ receptors are members of the family of pentameric ligand-gated ion channels. Each subunit has an extracellular, transmembrane, and intracellular domain. Only part of the intracellular domain structure has been solved, revealing it contains two α-helical segments; one, the MA helix, is an extension of M4, while the other, the MX helix, is formed from residues located close to the end of M3. This MX helix is in distinct locations in open and closed receptor structures, suggesting it may play a role in function. Here, we explore this hypothesis using functional responses of Ala-substituted mutant receptors expressed in HEK293 cells. The data show altering many of the MX residues results in a small decrease in EC_50_ (up to 5-fold), although in one (H232A) this is increased. Radiolabeled ligand binding on selected mutants showed no change in binding affinity, indicating an effect on gating and not binding. In addition, five mutations (P316A, V317A, P318A, D319A, and H323A) initially resulted in nonfunctional receptors, but the function could be rescued by coexpression with a chaperone protein, suggesting a likely role in assembly or folding. Examination of previously obtained MD simulation data shows that the extent of MX encompassed by membrane lipids differs considerably in the open and closed structures, suggesting that lipid–protein interactions in this region could have a major effect on channel opening propensity. We conclude that the MX helix can modulate the function of the receptor and propose that its interactions with membrane lipids play a major role in this.

## Introduction

Cys-loop receptors are part of the pentameric ligand-gated ion channel (pLGIC) superfamily, proteins that are critical for fast synaptic transmission in the central and peripheral nervous systems of both vertebrates and invertebrates and typified by the nicotinic acetylcholine receptor (nAChR).^1^ They can be either homomeric or (more usually) heteromeric. Each of the five subunits possesses a large extracellular, ligand-binding domain containing the eponymous Cys-loop (a 13 amino acid disulfide-bonded peptide loop), a channel-forming transmembrane domain, consisting of four membrane-spanning segments, M1–M4, and an intracellular domain. The intracellular domain is the least well understood, despite playing roles in receptor trafficking, single-channel conductance, and modulation.

**Figure 1:**
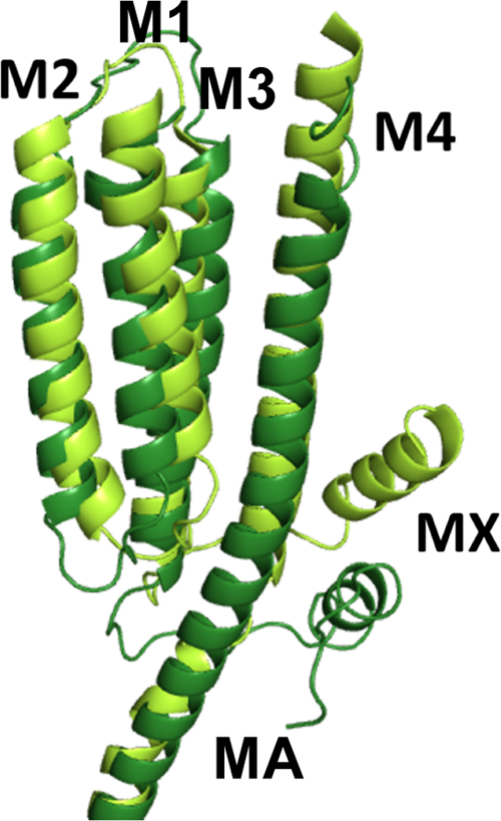
MX helix is in different locations in the resting (dark green, pdbid: 6BE1) and open (light green, 6DG8) 5-HT_3_R states. This indicates MX moves upwards and outwards relative to M4 when the receptor transitions from the resting to the open state. Specific residues alter positions by up to 18 Å.

The structure of the 5-HT_3_A homopentamer has been solved by both X-ray crystallography^2^ and cryo-electron microscopy (cryo-EM).^3,4^ These data reveal a largely β-sheet extracellular domain (ECD) and α-helical transmembrane domain (TMD), as expected for a pLGIC. Unusually, however, the structures also reveal some details of the intracellular domain (ICD), a domain that is frequently removed prior to structural examination; these data suggest that the ICD is mostly disordered, with two regions of α-helix, which have been named the MA and MX helices. The MA helix is effectively a cytoplasmic extension of M4, while the MX helix is close to the end of M3 and is located at the TMD/ICD interface adjacent to M4 (Figure 1).

As bacterial pLGICs do not have an ICD and replacement with a short peptide in vertebrate receptors does not ablate function,^5^ this region has not been well studied. Yet, it is increasingly becoming apparent that the ICD can have major effects, e.g., binding of some cytoplasmic molecules here can inhibit ion flux.^6^ A comparison of putative open and closed (resting) receptor conformations suggests that the MX helix moves significantly during channel opening: in the unbound (apo) state, it lies almost parallel to the plasma membrane, while when the receptor is open, it is displaced laterally by up to 18 Å (Figure 1) pulling a post-M3 loop outwards from the central axis of symmetry. This post-M3 loop extends away from the M4 helix, creating lateral portals (dimensions 16.0 Å × 11.4 Å) that would allow the passage of hydrated Na^+^ ions. These data suggest that the MX helix motion is correlated with channel opening and could play a role in modulating signal transduction.

**Figure 2:**
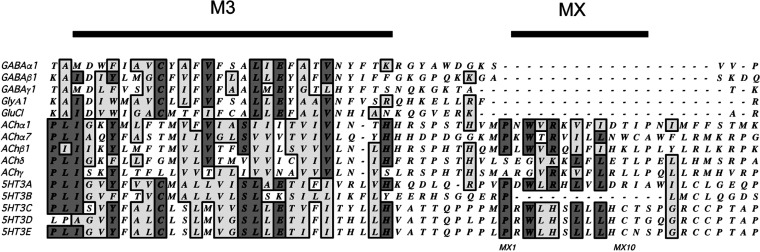
Sequence alignment of M3 and MX regions of anionic and cationic pLGICs shows that the MX helix may not be present in anionic receptors.

A further interesting feature of the MX helix is that it may be unique to the cationic pLGICs: no MX-like helical sections have yet been observed in glycine and GABA_A_ receptor structures, and, more compelling, an alignment of this region reveals some conservation of MX between the cationic 5-HT_3_ receptors and nACh receptors but no obvious similar region in anionic pLGICs (Figure 2).

We therefore decided to study the roles of the individual amino acids that contribute to the MX helix using Ala-scanning mutagenesis, and to use these data, combined with structural data, to consider if this region contributes to receptor function.

## Results

### Functional Characterization of Mutant and WT 5-HT_3_ R

To examine the function of wild-type (WT) and mutant 5-HT_3_A receptors, we transfected them into HEK293 cells and probed 5-HT-elicited responses in a Flexstation using membrane potential sensitive dye. Concentration–response curves for WT receptors revealed a 5-HT EC_50_ of 0.3 μM (pEC_50_ = 6.5 ± 0.04) and a Hill coefficient of 2.5 ± 0.5, consistent with previously published data.^7^

### Functional Characterization of Mutant 5-HT_3_ R

Five mutants did not respond to 5-HT following expression (P316A, V317A, P318A, D319A, and H323A). These were then coexpressed with RIC-3, a well-established chaperone of the 5-HT_3_A receptor,^8^ which has no significant effect on the EC_50_ of WT receptors. All of the nonfunctional receptors functioned when they were coexpressed with RIC-3; thus, our data suggest that Ala substitution of these residues in MX is deleterious to 5-HT_3_R expression.

**Figure 3:**
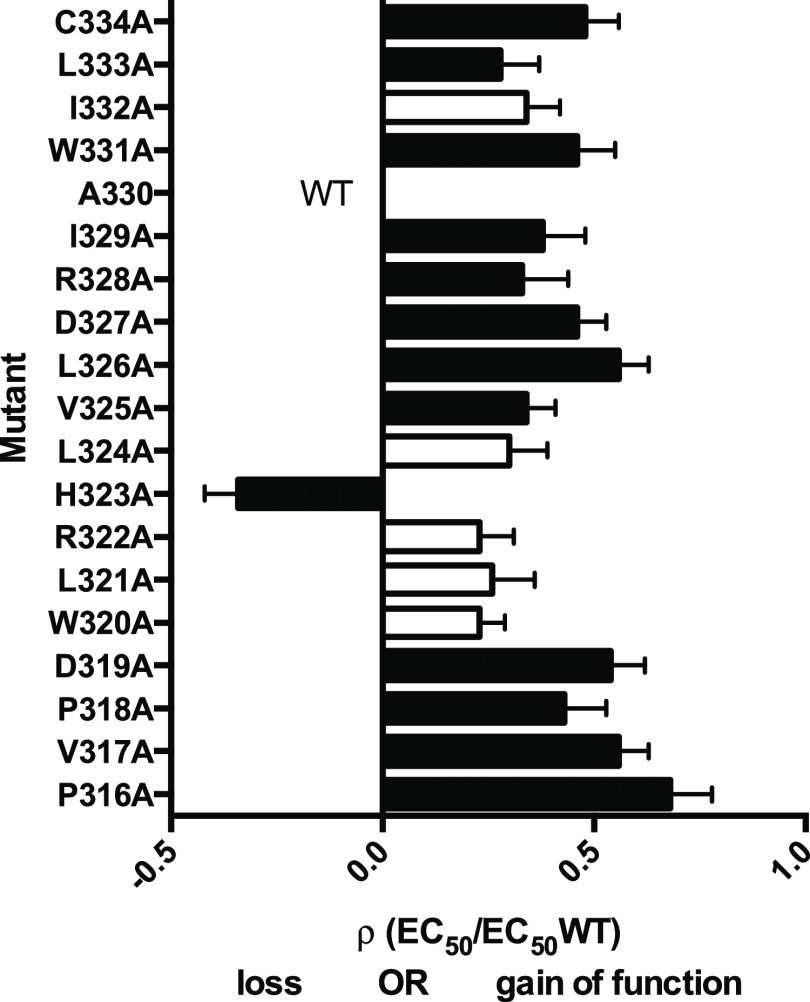
Plot showing many mutants result in a decrease of EC_50_ ( i.e., some gain of function) with one showing an increase ( i.e., some loss of function). Data from Table 1. Black = significantly different to WT, ANOVA with Dunnett’s multiple comparison test; *p* < 0.05.

**Figure 4:**
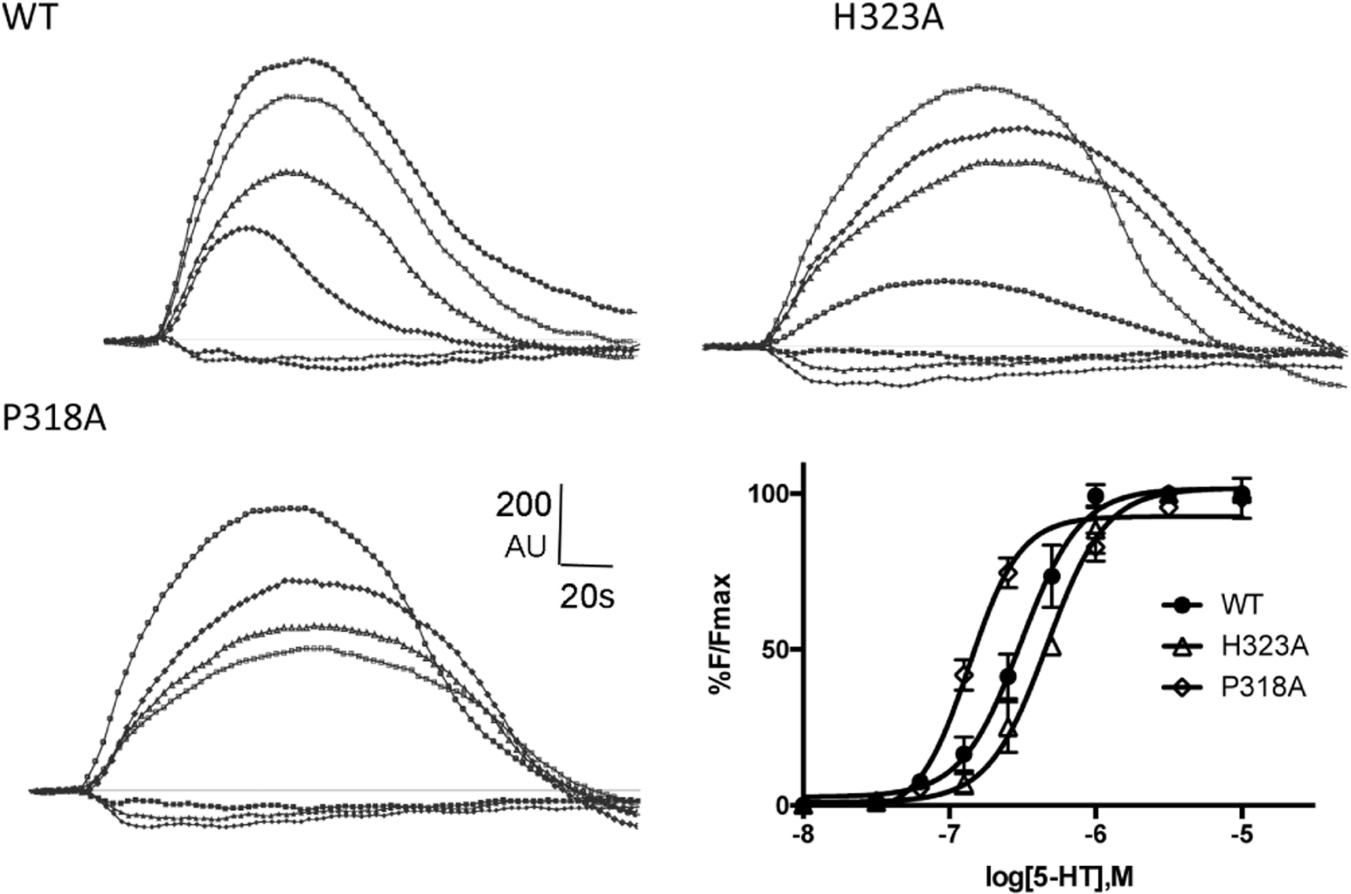
Typical fluorescent responses (F in arbitrary units, AU) of HEK293 cells expressing 5-HT_3_A receptors and stimulated at 20 s with a range of concentrations of 5-HT (0.01–3 μM), and concentration–response curves derived from these and similar data. See Table 1 for parameters.

The parameters determined for mutant receptors (with RIC-3 for those that were initially nonfunctional) reveal that Ala substitutions resulted in small changes in EC_50_s for 13/18 of the altered receptors (Table 1). All these changes decreased EC_50_s except for H323A, which caused an increase in EC_50_ (Figure 3). Typical Flexstation traces and concentration–response curves for this mutant, WT, and P318A (a mutant that causes a decrease in EC_50_) are shown in Figure 4.

**Table 1:** Functional Parameters for WT and Mutant 5-HT_3_R^a^

mutant	pEC_50_ (M)	EC_50_ (μM)	*n*_H_	*n*
WT	6.50 ± 0.04	0.32	2.5 ± 0.5	20
**WT**	6.52 ± 0.06	0.30	2.0 ± 0.2	8
**P316A**	7.18 ± 0.06^b^	0.07	1.7 ± 0.3	4
**V317A**	7.06 ± 0.05^b^	0.09	2.0 ± 0.4	8
**P318A**	6.93 ± 0.06^b^	0.12	1.4 ± 0.3	8
**D319A**	7.04 ± 0.04^b^	0.09	2.4 ± 0.5	4
W320A	6.73 ± 0.02	0.19	3.6 ± 0.4	4
L321A	6.76 ± 0.06	0.18	1.6 ± 0.2	4
R322A	6.73 ± 0.04	0.19	1.8 ± 0.2	4
**H323A**	6.16 ± 0.05^b^	0.69	2.4 ± 0.5	4
L324A	6.80 ± 0.04	0.16	3.3 ± 0.8	4
V325A	6.84 ± 0.03^b^	0.14	2.5 ± 0.3	4
L326A	7.06 ± 0.03^b^	0.09	3.2 ± 0.5	4
D327A	6.96 ± 0.03^b^	0.11	4.4 ± 1.2	4
R328A	6.83 ± 0.07^b^	0.15	1.7 ± 0.4	4
I329A	6.88 ± 0.06^b^	0.13	2.5 ± 0.8	4
W331A	6.96 ± 0.05^b^	0.11	2.3 ± 0.5	4
I332A	6.84 ± 0.04^b^	0.14	3.2 ± 1.0	4
L333A	6.78 ± 0.05	0.17	2.8 ± 0.8	4
C334A	6.98 ± 0.04^b^	0.11	3.4 ± 0.8	4

a Bold = data obtained when coexpressed with RIC-3. *p* = −log; pEC_50_s are used as they are normally distributed about the mean. Data = mean ± SEM.

b Significantly different to WT, ANOVA with Dunnett’s multiple comparison test; *p* < 0.05.

### Radiolabeled Ligand Binding

**Figure 5:**
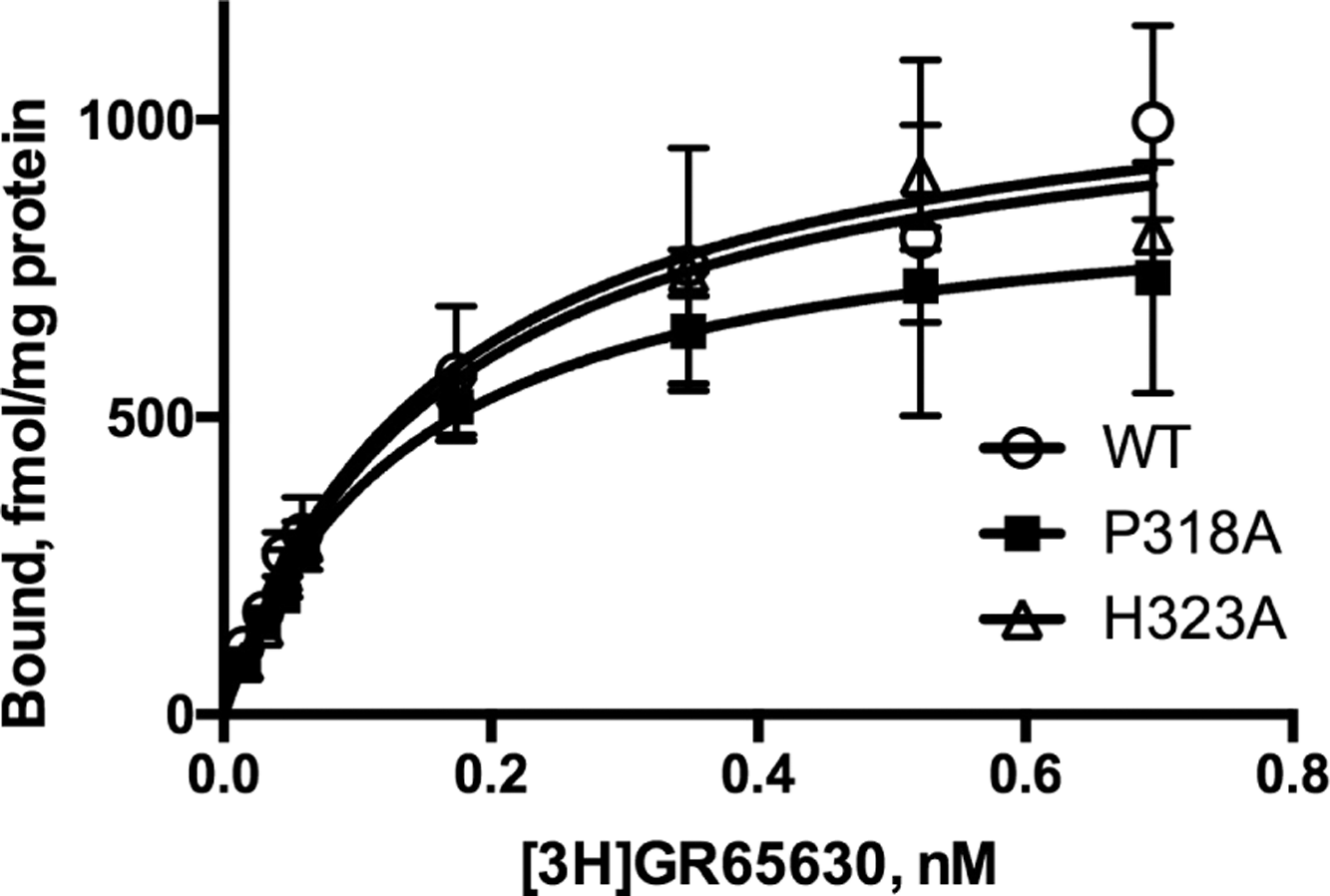
Example of radioligand binding curves from HEK cells expressing WT or mutant receptors. Parameters derived from such curves are similar: WT: *K*_d_ = 0.25 ± 0.1 nM, *B*_max_ = 1.1 ± 0.4 pmol/mg protein; P318A: *K*_d_ = 0.16 ±. 06 nM, *B*_max_ = 0.9 ± 0.2 pmol/mg protein; H323A: *K*_d_ = 0.18 ±. 02 nM, *B*_max_ = 1.0 ± 0.1 pmol/mg protein (data = mean ± SEM, *n* = 4).

EC_50_ values are useful measures to compare the effect of a mutation on the function of the protein, but they do not reveal whether the mutation affects binding or gating. One method to explore this is to perform radiolabeled ligand binding to examine the parameters associated with the binding site. Our data (Figure 5), using a subset of mutant receptors, revealed that the binding affinity of mutant and WT receptors was not significantly different. These data indicate that the changes in EC_50_ we observed were likely due to changes in gating.

### Further Characterization of Residue P318

**Figure 6:**
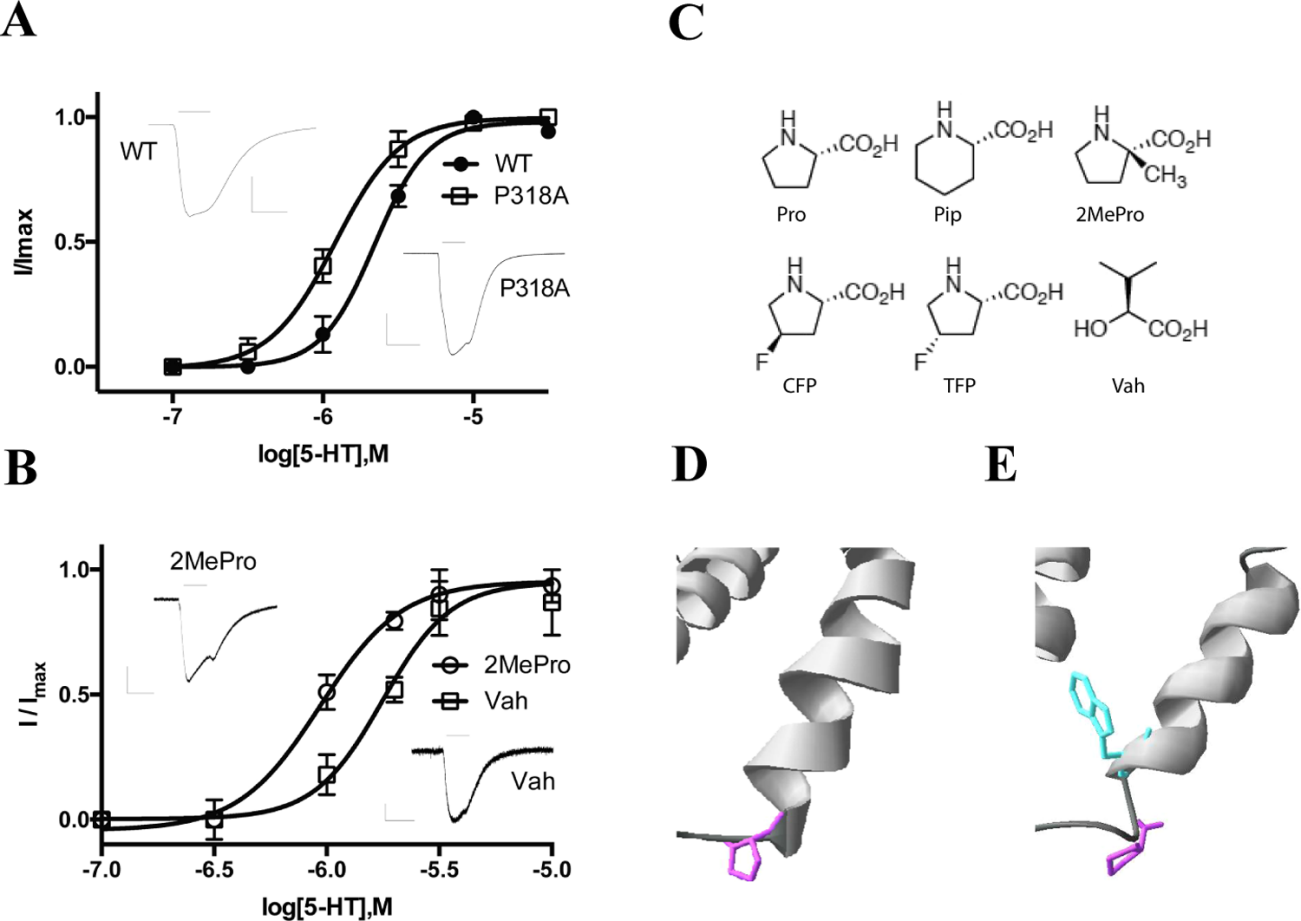
(A, B) Concentration–response curves and typical traces of WT and mutant 5-HT_3_ R substituted with (A) canonical or (B) noncanonical amino acids. Parameters derived from these data are in Table 2. Example traces elicited by 10 μM 5-HT are also shown; scale bars = 10 s and 2 μA (WT and P318A), 0.2 μA (2MePro), and 0.1 μA (Vah). (C) Structures of the noncanonical amino acids. (D,E) Structural data show that P318 (purple) is located at the start of the MX helix in the 6DG8 (open) structure (D) but W320 (cyan) has this position in the 6BE1 (resting) structure (E).

P318 is located at the start of the MX helix (Figure 6) and a P318A substitution was typical in causing a decrease in EC_50_; we therefore decided to further explore the role of this residue using an expression in oocytes with both canonical and noncanonical amino acids. The data (Figure 6A,B, Table 2) show that the small decrease in EC_50_ observed with P318A-containing receptors in our HEK cell experiments was maintained when using oocyte expression, and a similar small decrease was seen with a P318V substitution but not with a P318G mutation. For the noncanonical amino acid experiments, EC_50_s were similar to WT for those substituted with *cis*-4-fluoroproline (CFP), *trans*-4-fluoroproline (TFP), and α-hydroxyvaline (Vah), but again we observed a decreased EC_50_ when P318 was substituted with pipecolic acid (Pip) or 2-methylproline (2MeP). These noncanonical amino acids are all proline analogues that can probe the unusual properties of proline; these properties include an increased propensity for a cis peptide bond, a decreased hydrogen bonding capacity, and a kink or bulge in an α-helix. CFP and TFP are close analogues of Pro, but the incorporation of a single F in a *cis* or *trans* configuration results in different cis peptide bond propensities (TFP = 12%, CFP = 28%^9^); thus our data (no difference) suggest that this aspect of Pro plays no role here. However, a different shape or size at position 318, as in Pip (differs from Pro in having a larger ring) or 2MeP (a methyl group), results in a similar change in EC_50_ to that we observed with Ala and Val. In contrast, Vah substitution (generating receptors with a backbone ester in place of an amide) suggests that the decreased hydrogen bonding capacity allows Vah to maintain a WT-like EC_50_. Overall, these data are not incompatible with a more bulky residue at P318 being deleterious to function, and our speculative hypothesis is that this impairs the flexibility of this region, which needs to rapidly shift between α–helical (in the open state, Figure 6D) and non-α-helical (in the closed state, Figure 6E).

**Table 2:** Functional Parameters for WT and Mutant 5-HT_3_R from Oocyte Experiments^a^

mutant	pEC_50_ (M)	EC_50_ (μM)	*n*_H_	*n*
WT	5.66 ± 0.03	2.2	2.5 ± 0.3	8
P318A	5.92 ± 0.03^b^	1.2	2.0 ± 0.3	4
P318G	5.80 ± 0.06	1.5	2.3 ± 0.6	4
P318V	5.97 ± 0.04^b^	1.1	2.2 ± 0.6	4
Pro	5.81 ± 0.02	1.5	2.4 ± 0.1	8
CFP	5.89 ± 0.03	1.3	2.1 ± 0.3	4
TFP	5.99 ± 0.03	1.0	2.8 ± 0.4	4
Pip	6.04 ± 0.03^b^	0.9	2.7 ± 0.5	4
2MeP	6.03 ± 0.02^b^	0.9	2.4 ± 0.5	4
Vah	5.89 ± 0.05	1.3	2.2 ± 0.6	4

a Data = mean ± SEM.

b Significantly different to WT or Pro recovery (for noncanonical amino acids), ANOVA with Dunnett’s multiple comparison test; *p* < 0.05.

### In Silico Studies

To explore the potential for lipid interactions with MX, we examined and compared the 800 ns simulations of two 5-HT_3_R structures taken from MemProtMD.^10^ The currently available high-resolution 5-HT_3_R structures are assigned a range of states mainly based on their pore diameters. To avoid variations due to different methods and preparation protocols, we selected two structures from a single group, i.e., prepared with similar equipment/protocols, and compared a structure classed as resting (6BE1) with one classed as open (6DG8).

**Figure 7:**
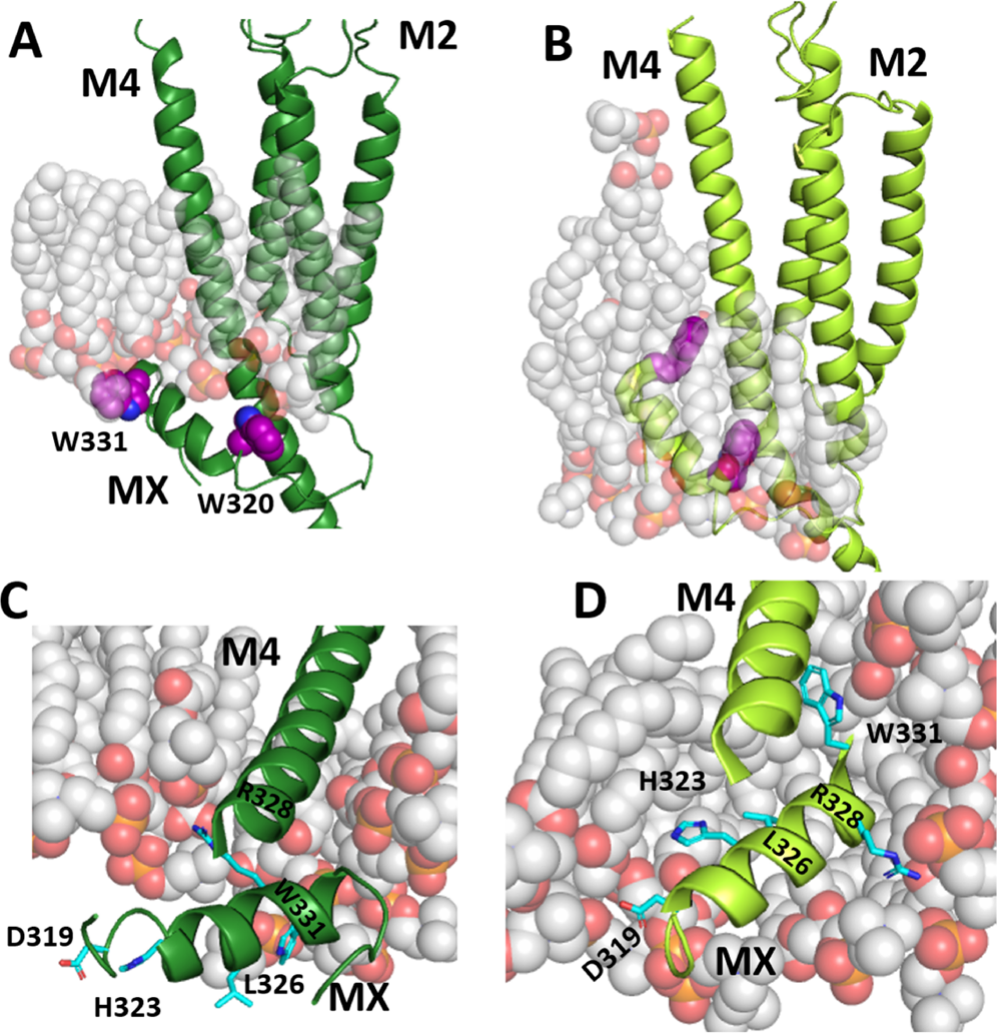
Snapshots following an 800 ns simulation of 6BE1 (left) and 6DG8 (right) embedded in a phosphatidylcholine membrane.^10^ Panels (A) and (B) show the MX and transmembrane domain of one subunit, as well as the lipids within 5 Å of MX. Trps at each end of MX are shown for orientation. Panels (C) and (D) show the MX and M4 of one subunit, as seen from an oblique intracellular angle, highlighting functionally relevant MX residues.

The difference in the position of MX in the 6BE1 and 6DG8 structures (Figure 1) is maintained even at the end of the 800 ns simulations (Figure 7). Panels A and B show how MX moves from below the membrane to completely within it: in the 6BE1 structure (Figure 7A), the tip of the MX is contacting the polar headgroups of the lower lipid bilayer and the rest of MX is intracellular. In the 6DG8 structure (Figure 7B), MX is fully embedded within the membrane, with the tip even reaching some upper leaflet lipids and the bottom/hinge area now contacting the intramembrane sides of the polar headgroups. Panels C and D show the positions of some residues we identified as affecting EC_50_ (Table 1, Figure 2) and how their environment differs between the open and closed states. D319, H323, and L326 go from being entirely cytoplasmic to fully embedded in the membrane—D319 and to some degree H323 interacting with polar headgroups and L326 thrust into the hydrophobic lipid tails. This also shows the major rotation that the distal end of MX undergoes, with L326, R328, and W331 in very different orientations with respect to M4 in the two different channel states. Unfortunately, we can draw no conclusions about specific lipid interactions under physiological conditions from these simulations, as they were performed in phosphatidylcholine-only membranes. Nevertheless, the images show that a number of MX residues move between hydrophilic and hydrophobic environments, and so this region could be an important contributor to channel opening/closing dynamics.

## Discussion

The aim of this study was to determine whether the MX helix residues play a role in the 5-HT_3_R function by testing the function and expression of receptors when each individual MX residue was mutated to alanine. The data indicate that five of the residues are likely involved in assembly and/or surface expression of the receptor, as a chaperone is required for function. In addition, a large number of the mutant receptors resulted in a small decrease in EC_50_, indicating a gain of function, with one showing an increase (Figure 3). Radiolabeled binding studies showed no change in the ligand affinity at the binding site, suggesting that the mutations resulted in a change in gating. Images showing the position of lipids relative to MX following 800 ns MD simulations are consistent with the movement of this helix into the membrane when the pore opens. The data, which are discussed in more detail below, suggest that residues in this α-helix can contribute to receptor function and may be critical for transducing the effects of changes in the ICD (such as when a modulator binds) to the pore.

### MX as a Region Involved in Receptor Expression

**Figure 8:**
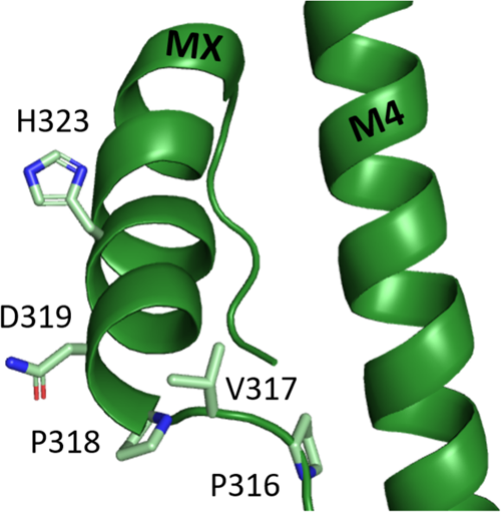
Residues of the MX helix where an Ala substitution ablated function, which was restored by RIC-3. The charged residues D319 and H323 could potentially interact with lipid headgroups.

Previous studies in the nAChR have shown that the equivalent region in these receptors regulates receptor assembly and trafficking.^11^ Our data show that five mutants, which did not initially respond to 5-HT yet did function when coexpressed with RIC-3, support a similar role here. RIC-3 is a well-established chaperone of the 5-HT_3_A receptor, acting via interactions with the MX helix to facilitate receptor assembly.^6,8,24^ Our data suggest that Ala substitution of certain residues in MX impedes assembly in the absence of RIC-3. The affected residues (Figure 8) include D319 and H323, which are charged residues that face away from M4, and we speculate that these residues may be important for interaction with lipid head groups to guide the correct insertion of this region of the receptor into the bilayer.

### Mutations Resulting in a Gain of Function

Most of the Ala-substituted receptors showed small decreases in EC_50_s, which the radiolabeled binding data suggest are not due to a change in binding affinity, and thus may reflect enhanced gating of the receptor. This is reminiscent of data obtained from studies of the M4 region of some pLGICs, e.g., in ELIC, substitution with Ala resulted in small decreases in EC_50_s,^12^ supporting previous data that suggested that this helix contributes to their function.^13−16^ In the case of ELIC, it was proposed that closer packing of M4 with adjacent transmembrane helices and/or greater conformational flexibility is advantageous. Considering these as potential explanations for our MX data, closer helical packing is unlikely as there is a limited association of MX with adjacent helices. Greater conformational flexibility is a possibility, as there is a change not only in the location but also in the length of the helix between the open and closed states of the receptor (Figure 1). A further explanation, which we consider more likely, is a change in the association with adjacent lipids: previously performed simulations show very different potential MX–lipid interactions in the resting and open states, and we used a snapshot from these simulations to show the positions of lipids close to MX (Figure 7). These images show that lipids would be able to interact with all the residues in MX in the open state but only some in the closed state, i.e., lipid interactions could change significantly during gating. This could explain our data, as any changes to MX residues could alter their lipid interactions and hence modify the stability of MX in the open and/or closed state. Other routes that modify MX, such as a change in the structure of the loops on either side of this helix, could have a similar effect. Indeed, we speculate that in vivo the MX helix may be the route by which modifications to the M3-M4 loop are transduced to the pore, as this region has a number of motifs including phosphorylation sites and binding sites for regulatory and cytoplasmic signaling molecules.

We cannot yet determine whether or which lipids interact specifically with particular MX residues, but studies in other pLGIC have shown certain lipids are integral to their structure and/or function.^17−19^ Cholesterol, for example, has long been known to be important in the nAChR function,^20^ and in the α4β2 nAChR, cholesterol molecules are bound on the intracellular side of the TM domain.^21^ Recent studies in ELIC show an integral bound PE lipid, which contributes to the agonist response.^17^ The ELIC lipid-binding site is partly shaped by a Pro-induced kink in M4, which is also apparent in GABA and glycine receptors but not in cation-selective pLGICs. An alternative conformation of M4 observed in ELIC suggests that M4 can exist in more than one orientation, swiveling via this kink, and the particular location of the part (or all) of the helix may regulate lipid interactions and thereby channel function. Given that this kink does not occur in the 5-HT_3_R M4 and MX may not be present in anion-selective channels, a speculative hypothesis is that MX in cation-selective receptors could perform a similar lipid-interacting function to that of M4 in anion-selective receptors.

### Using the Structural Data to Understand the Role of MX

**Figure 9:**
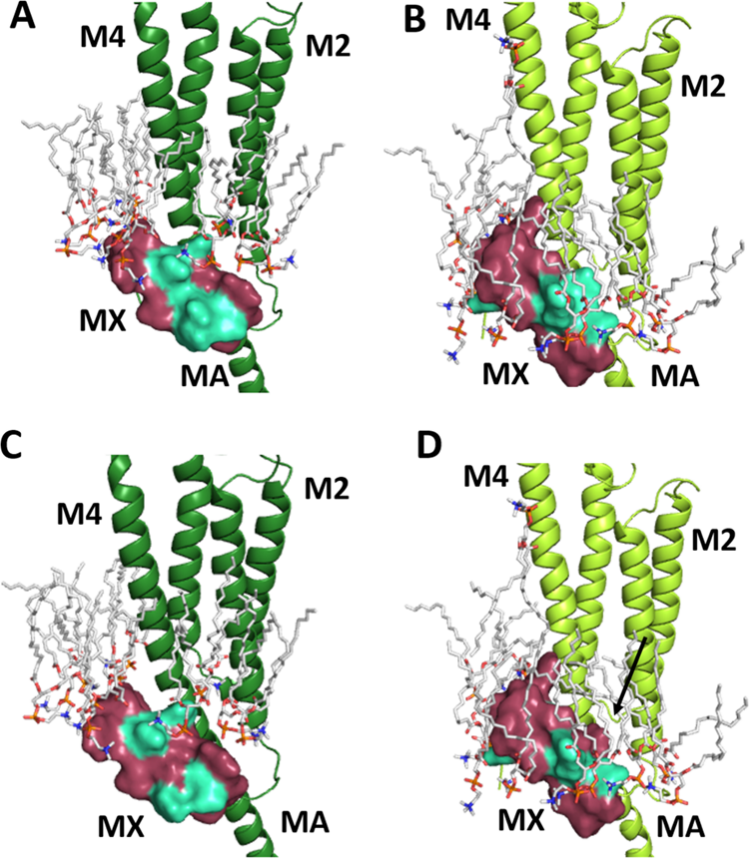
Structure of resting (A, C 6BE1) and open (B, D 6DG8) 5-HT_3_R MX helices (space fill) show different patterns of hydrophobicity when viewed from the membrane. Magenta = hydrophobic residues, blue = hydrophilic residues. (A, B) WT; (C, D) H323A, with an arrow indicating the mutation site in panel (D).

The sequence alignment (Figure 2) suggests that there are a number of relatively conserved residues in the MX helix: a Pro (P318 in the 5-HT_3_R) at the start of the helix, which we will define as position MX1, a Trp at MX3, a positive residue at MX5, and three hydrophobic residues followed by a charged residue at position MX9 and/or MX10. Thus MX has a distinct hydrophobicity pattern. If the location and relative orientation of MX differs in the resting and open states, as it does in the 6BE1 and 6DG8 structures, then the hydrophobic pattern facing outwards from the receptor differs: in the resting state, there is a patch of hydrophobic residues at the C terminal end of the helix, while there is a stripe of hydrophobicity along the whole of MX in the open state (Figure 9). These different patterns likely contribute to the ability of MX to move as there will be different propensities of MX to interact with lipids in the different states.

We note that the decreases in EC_50_ we observed are for Ala mutant residues, which are mostly clustered close to P318 or at the N-terminal region of the helix. Ala substitutions are likely to make MX more flexible and less bulky, which could facilitate its movement into the membrane and concomitant channel opening. H323A is the only MX mutation to increase EC_50_, suggesting that the significant alteration to the hydrophobicity profile (Figure 9) interferes with specific interactions with local lipids, which favor the entry of MX into the membrane. Further studies are needed to explore these possibilities.

## Conclusions

In conclusion, our data contribute to understanding the role of MX in the 5-HT_3_R function. This region and indeed the whole M3-4 loop are a bit of an enigma, as, while complete removal does not prevent function, binding of some chaperones can. Here, we show that changes to MX alter the function and suggest that this may be linked to changes in lipid interactions that are important for the movement of MX into the membrane upon channel opening. Further studies, such as those with partial agonists, different lipids, single-channel data, and/or structural studies (such as those that have recently shown details of the nAChR ICD^25^) should help clarify how MX performs its role.

## Methods

### Cell Culture

Human embryonic kidney (HEK) 293 cells were maintained in 90 mm tissue culture plates at 37 °C and 7% CO_2_ in a humidified atmosphere. They were cultured in Dulbecco’s Modified Eagle’s Medium/Nutrient Mix F12 (1:1) with GlutaMAX I media (Life Technologies, Paisley, U.K.) containing 10% HyClone fetal calf serum (GE Healthcare). For FlexStation studies, cells were transfected using polyethylenimine (PEI; Polysciences). Cells were then transferred to poly-l-lysine (Cultrex)-coated 96-well plates and allowed to adhere overnight before use.

### FlexStation Studies

The methods were as described previously.^7^ In brief, fluorescent membrane potential dye (Membrane Potential Blue kit, Molecular Devices) was diluted in Flex buffer (10 mM HEPES, 115 mM NaCl, 1 mM KCl, 1 mM CaCl_2_, 1 mM MgCl_2_, and 10 mM glucose, pH 7.4) and added to each well. Following incubation at 37 °C for 30 min, fluorescence was measured in a FlexStation 3 (Molecular Devices) at 2 s intervals for 200 s. 5-HT (Sigma) was added to each well after 20 s. Data were normalized to the maximum Δ*F* and analyzed using Prism (GraphPad Software Inc.).

### Molecular Biology

Site-directed mutagenesis was performed using the Stratagene QuikChange protocol to generate the appropriate codon. For noncanonical amino acid mutants and conventional mutants generated by nonsense suppression, the site of interest was mutated to the TAG stop codon. Plasmids were linearized with the SbfI restriction enzyme, and receptor mRNA was prepared by *in vitro* runoff transcription using the Ambion T7 mMessage mMachine kit.

Hydroxy or amino acid-dCA conjugates were enzymatically ligated to truncated 74mer THG73 tRNA as previously described.^22^ The 74mer tRNA was prepared using the Ambion T7MEGAshortscript kit. Deprotection of the NVOC group on the tRNA-amino acids was carried out by photolysis for 5 min on a 300 W high-pressure Hg arc lamp with WG-335 and UG-11 filters immediately prior to injection.

### Oocyte Preparation and RNA Injection

Stage V–VI oocytes of *Xenopus laevis* were harvested and injected with RNAs as described previously.^22^ For nonsense suppression experiments, each cell was injected with 50–100 ng each of receptor mRNA and appropriate tRNA approximately 48 h before recording.

For wild-type experiments and conventional mutants, each cell received a single injection of 1–25 ng of receptor mRNA approximately 24 h before recording. Injection volumes for each injection session were 50–100 nL per cell.

Wild-type recovery conditions (injecting tRNA charged with the appropriate amino acid to regenerate a wild-type channel *via* nonsense suppression at a TAG stop codon) were used alongside mutant nonsense suppression as a positive control.

### Electrophysiology

Two-electrode voltage clamping of *Xenopus* oocytes was performed using standard electrophysiological procedures as previously described^22^ using either a GeneClamp 500 amplifier or an OpusXpress system (Axon Instruments, Inc., Union City, CA). All experiments were performed at 22–25 °C. 5-HT (Sigma) was diluted in ND96 and delivered to cells via a computer-controlled perfusion system. Glass microelectrodes were backfilled with 3 M KCl and had a resistance of approximately 1 MΩ. The holding potential was −60 mV unless otherwise specified. Concentration–response curves and parameters were obtained using Prism software (GraphPad, PRISM, San Diego, CA).

### Radioligand Binding

This was undertaken as previously described.^23^ Briefly, transfected HEK293 cell membranes were incubated in 0.5 ml of HEPES buffer containing the 5-HT_3_ receptor antagonist [^3^H]GR65630 to label cell surface receptors. Nonspecific binding was determined using 1 μM quipazine. Data were analyzed by iterative curve fitting using Prism. Values are presented as mean ± SEM.

### In Silico Studies

To explore possible lipid interactions with MX, snapshots from the end of an 800 ns simulations of 6BE1 and 6DG8 were downloaded from MemProtMD,^10^ an online database of coarse-grained molecular dynamics simulations of membrane proteins in lipid bilayers. Structures were viewed and images were prepared using Pymol (https://pymol.org).

## Abbreviations Used

5-HT: 5-hydroxytryptamine
nACh receptor: nicotinic acetylcholine
GABA: γ-aminobutyric acid
HEK: human embryonic kidney
AChBP: acetylcholine-binding protein
